# Thyroid Hemiagenesis: A Longitudinal Case Report of Dynamic Thyroid Function

**DOI:** 10.7759/cureus.97418

**Published:** 2025-11-21

**Authors:** Afnan H AlSayed, Alanood A Alqerainees, Abdullah K Alhwimani

**Affiliations:** 1 College of Medicine, Alfaisal University, Riyadh, SAU; 2 Family Medicine and Polyclinics, King Faisal Specialist Hospital and Research Centre, Riyadh, SAU

**Keywords:** congenital thyroid anomalies, pediatric endocrinology, rare case report, subclinical hypothyroidism, thyroid function, thyroid hemiagenesis, ultrasound thyroid, unilateral thyroid

## Abstract

Thyroid hemiagenesis is a rare congenital anomaly characterized by the absence of one thyroid lobe. Most cases are asymptomatic and diagnosed incidentally. It is an important condition to recognize clinically, as the compensatory function of the remaining lobe may not be sufficient over time and may result in thyroid dysfunction. A 15-year-old female was referred for routine assessment because of a paternal family history of hypothyroidism. Physical examination revealed an enlarged right thyroid lobe, and ultrasonography confirmed congenital absence of the left lobe. Laboratory tests revealed initial subclinical hypothyroidism, which normalized after thyroxine therapy. A long-term seven-year follow-up showed that thyroid function had remained stable without pharmacological intervention because of effective compensatory hypertrophy of the right lobe. The child remained asymptomatic with continued normal growth and development. This unique case of thyroid hemiagenesis in a 15-year-old female, where the remaining thyroid lobe maintained normal thyroid function with normal thyroid function tests despite the structural anomaly, highlights the importance of thorough assessment and long-term follow-up. While compensatory hypertrophy can keep thyroid function normal, subclinical hypothyroidism may still develop over time. The asymptomatic nature of this patient, in contrast to some cases reporting mild symptoms or subclinical hypothyroidism, underscores the need for ongoing monitoring to detect potential future dysfunction. Familial thyroid history may also provide valuable insights for management.

## Introduction

Thyroid hemiagenesis is a rare congenital anomaly resulting from disturbed embryological development, resulting in the absence of one thyroid lobe [1]. The condition must be differentiated from other thyroid dysgenesis, including hypoplasia (underdevelopment of a lobe), aplasia (complete absence of the gland), and ectopic thyroid tissue, which may be located in places such as the lingual or mediastinal position. There was no ectopic thyroid tissue detected on imaging, and it was ruled out clinically in our patient. The thyroid gland develops during the third week of gestation and completes its descent into the neck by the seventh week [2]. Thyroid hemiagenesis most commonly involves left thyroid lobe absence in 70-80% of reported cases, with right-sided hemiagenesis being rarer but possibly having a higher risk of thyroid dysfunction [3,4]. Compensation for the remaining lobe can maintain normal hormonal output. Some may present with thyroid dysfunction, such as hypothyroidism, hyperthyroidism, or autoimmune thyroid disease, if compensatory mechanisms fail [4]. The presence of familial predisposition or other risk factors can further affect the clinical course of thyroid hemiagenesis [3,5]. A 15-year-old girl was diagnosed with thyroid hemiagenesis during a routine check-up due to a family history of hypothyroidism.

## Case presentation

A 15-year-old female was seen for a routine visit with a significant paternal history of hypothyroidism. She was asymptomatic, with no fatigue, weight gain, cold intolerance, or neck swelling. She did not have any significant medical history; her growth and developmental milestones were also within normal. She had three younger female siblings who were all normal concerning thyroid anatomy and function. The parents were not consanguineous.

Examination showed a mildly enlarged right lobe; the left lobe was non-palpable. It was non-tender and not associated with dysphagia, dysphonia, or dyspnea. There was no cervical lymphadenopathy, eye changes, or skin changes suggestive of autoimmune thyroid disease. Systemic examination was normal; there was no dextrocardia.

Follow-up from 2017 to 2025 revealed that she grew undisturbed. In 2017, at the age of 15 years, her height was 160 cm, and her weight was 59 kg. Her height did not change afterward, while her weight gradually increased from 59 kg in 2017 to 70 kg by 2025, matching a gradual rise in body mass index (BMI) from 23.34 kg/m² (normal range) to 27.34 kg/m² (overweight range). The gradual weight gain matched her transition into and through early adulthood. No clinical symptoms of hypothyroidism, such as fatigue, intolerance to cold, or significant complaints in relation to the increase in weight, were reported accompanying the increased BMI. This indicated a well-compensated thyroid function despite congenital thyroid hemiagenesis. The patient’s stable growth and normal developmental milestones indicated the effectiveness of compensatory hypertrophy in her right thyroid lobe, emphasizing the need for monitoring BMI and thyroid anomalies.

**Figure 1:**
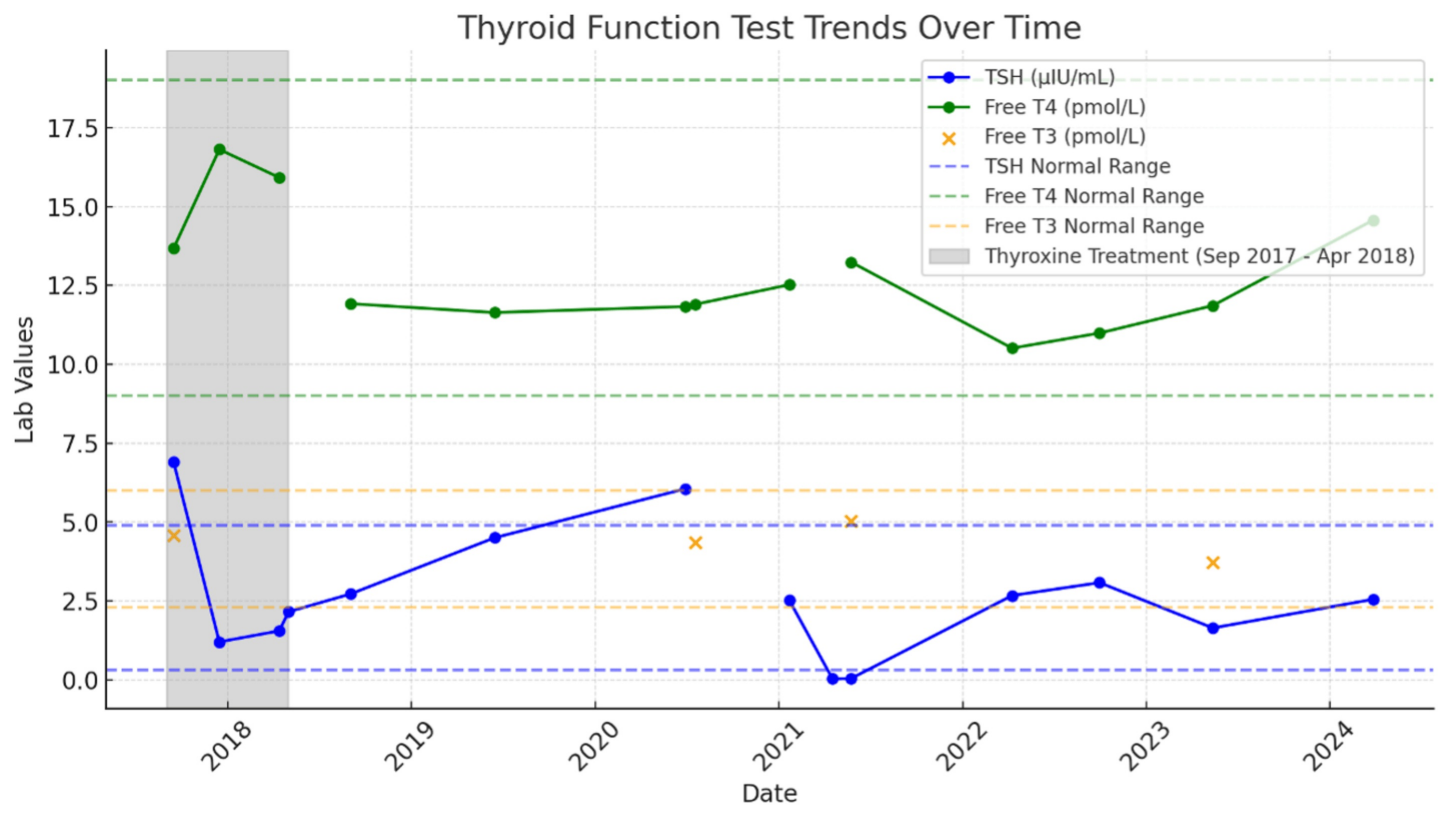
Timeline of the laboratory results. Plot of thyroid function tests from 2017 to 2024, illustrating trends in TSH (blue), free T4 (green), and free T3 (orange). The shaded (grey) zone represents the introduction of daily 25 µg levothyroxine replacement therapy (September 2017 to April 2018). TSH = thyroid-stimulating hormone; Free T4 = free thyroxine; Free T3 = free triiodothyronine

The patient’s thyroid function showed dynamic changes following daily thyroxine therapy of 25 µg from September 2017 to April 2018, initially improving with a period of overcorrection (Figure 1). Long-term follow-up showed stable function, good compliance, and normalization of function likely attributed to the right lobe compensatory function (Table 1). The patient remained asymptomatic and continued annual follow-ups.

**Table 1:** Timeline of thyroid function and clinical course (2017–2024). Serial follow-up of thyroid function documented dynamic changes over seven years. In 2017, the patient initially presented with subclinical hypothyroidism, requiring short-term levothyroxine therapy. This was followed by normalization and later mild TSH variability, reflecting compensatory hypertrophy of the right lobe. A transient episode of hyperthyroidism in 2021, consistent with thyroiditis, spontaneously resolved. Thyroid function stabilized from 2022 to 2024, indicating successful long-term compensation by the right lobe. TSH = thyroid-stimulating hormone; Free T4 = free thyroxine

Date	Clinical status	TSH (µIU/mL)	Free T4 (pmol/L)	Notes
September 2017	Asymptomatic, subclinical hypothyroidism	6.91	13.67	Started levothyroxine 25 µg daily
April 2018	Asymptomatic, overcorrected	1.55	15.92	Levothyroxine discontinued
2019–2020	Stable	4.5–6.0	~11–12	Compensated right lobe
2021	Transient hyperthyroidism	0.029–0.041	13.2	Suspected thyroiditis, self-resolved
2022–2024	Normal, asymptomatic	1.6–3.0	11–14	Stable compensatory function

**Figure 2:**
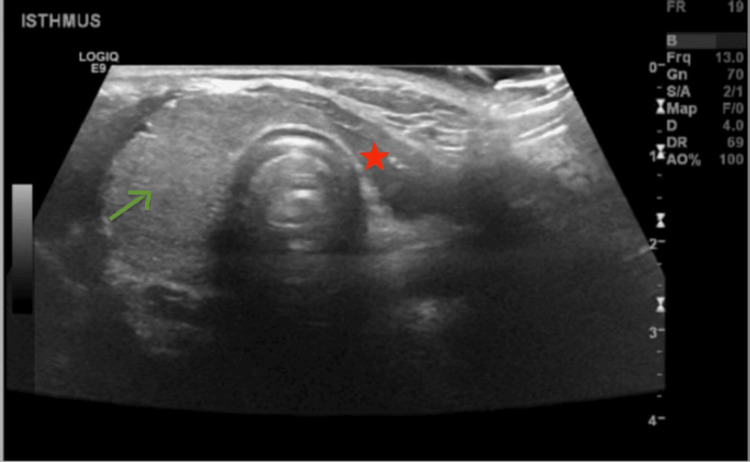
Ultrasound image showing absence of the left thyroid lobe and compensatory hypertrophy of the right lobe in a 15-year-old female with thyroid hemiagenesis. Thyroid ultrasound (longitudinal section of anterior neck) showing midline-located thyroid gland demonstrating absence of the left thyroid lobe and compensatory hypertrophy of the right lobe in a 15-year-old female with thyroid hemiagenesis. The isthmus was present and measured 0.27 cm. A scale bar is included. Green arrow: enlarged right lobe.

Due to the physical examination findings of an enlarged right thyroid lobe, an ultrasound was requested (Figure 2). A summary of thyroid function and clinical course is given in Table 2. The patient initially required temporary levothyroxine therapy but eventually had stable function by means of compensatory hypertrophy of the right lobe, with only a transient thyroiditis disease in 2021.

**Table 2:** Laboratory investigations conducted over several years revealing dynamic changes in thyroid function. TSH = thyroid-stimulating hormone; Free T4 = free thyroxine; Free T3 = free triiodothyronine; Anti-TPO = anti-thyroid peroxidase antibodies; TRAbs = thyroid-stimulating hormone receptor antibodies. Reference ranges: TSH: 0.4–4.0 µIU/mL, free T4: 10–25 pmol/L, free T3: 3.5–6.5 pmol/L (values may vary slightly by lab).

Date	Free T4 (pmol/L)	Free T3 (pmol/L)	TSH (µIU/mL)	Anti-TPO (IU/mL)	TRAbs (IU/L)
15/9/2017	13.67	4.57	6.91	-	-
15/12/2017	16.81	-	1.20	-	-
13/4/2018	15.92	-	1.55	-	-
2/5/2018	-	-	2.1562	4.7	<1
2/9/2018	11.92	-	2.72	-	-
15/6/2019	11.64	-	4.5	-	-
28/6/2020	11.83	-	6.052	-	-
18/7/2020	11.90	4.35	-	1.20	-
21/1/2021	12.52	-	2.523	-	-
17/4/2021	-	-	0.029	-	-
24/5/2021	13.24	5.03	0.041	-	-
9/4/2022	10.51	-	2.67	-	-
30/9/2022	10.99	-	3.078	-	-
13/5/2023	11.86	3.71	1.641	-	-
27/3/2024	14.57	-	2.55	-	-

The right lobe was enlarged with a heterogeneous texture but no nodules. The left lobe was absent, indicating congenital absence. The right lobe measured 5.04 × 1.68 × 1.68 cm, and the isthmus measured 0.27 cm, with average vascularity noted. Bilateral cervical lymph nodes were observed, with benign characteristics: right cervical region (0.83 × 0.34 cm, 0.70 × 0.35 cm) and left cervical region (0.59 × 0.25 cm, 0.75 × 0.25 cm). No compromise of the great vessels was seen. The impression was a congenital absence of the left thyroid lobe, an enlarged right lobe with possible thyroiditis, and non-specific cervical lymphadenopathy bilaterally.

## Discussion

Szczepanek-Parulska et al. reported that thyroid hemiagenesis, which is a congenital defect noted in approximately 0.05% of the population, results in compensatory hypertrophy and is usually hard to diagnose and treat. The thyroid gland, which starts development in the third week of gestation, can be influenced by genetic, environmental, or combined factors to develop anomalies such as thyroid hemiagenesis [3].

Thyroid hemiagenesis usually presents with the absence of the left thyroid lobe. It has been reported that in approximately 70-80% of all reported cases of hemiagenesis, the left thyroid lobe is affected, while it is more seldom that the right lobe is absent. Lateralization, as stated above, has also been shown through some studies, including the findings from Szczepanek-Parulska et al., where in 78% of the cases, there was an absence of the left lobe [1]. Further solidifying these results, Suzuki et al. reported that the most frequent form is indeed left-sided hemiagenesis, with few of these anomalies being on the right side [2]. In our case, the patient had the typical pattern of left thyroid lobe agenesis, as reported in the above statistics.

The remaining thyroid lobe in patients with thyroid hemiagenesis usually undergoes compensatory hypertrophy to preserve normal thyroid function. In our case, thyroid function remained within normal limits over successive years, suggesting that the compensatory mechanism effectively maintained hormone production. However, long-term dependence on a single lobe raises concerns about potential complications.

Some studies indicated that patients with thyroid hemiagenesis are at a higher risk for thyroid dysfunction, especially when the remaining lobe is under stress. According to a study by Unar et al., goiter can develop as the compensatory lobe enlarges, potentially leading to dysfunction [6]. Similarly, Khodak et al. and Kaba et al. reported that these patients may have an increased risk of autoimmune thyroid diseases or nodules due to the heightened functional burden [5,7]. Therefore, ongoing monitoring is essential to detect early signs of thyroid dysfunction, even in asymptomatic cases.

The patient’s thyroid function tests showed fluctuation in thyroid function test parameters, including thyroid-stimulating hormone (TSH) levels, free thyroxine (T4), and free triiodothyronine (T3) levels. From 2017-2018, elevated TSH indicated subclinical hypothyroidism, leading to temporary treatment with levothyroxine, which was stopped because the TSH levels returned to normal. Between 2018 and 2020, mild elevation followed stabilization, and, in 2021, transient hyperthyroidism, indicating subacute thyroiditis, but thyroid function normalized without pharmacological intervention between 2022 and 2024.

Our case of thyroid hemiagenesis shares similar unique features with several cases reported in the literature. For instance, a case reported by Szczepanek-Parulska et al. was of a young female patient with isolated thyroid hemiagenesis without any associated systemic abnormality [3]. Similar to our patient, she did not have any other congenital anomalies or a family history of thyroid diseases, making her an unusual case, as most cases of thyroid hemiagenesis described have other systemic associations. This further emphasizes the rarity of our case, where thyroid hemiagenesis was an isolated finding with no complicating conditions. In addition, Suzuki et al. noted that hypertrophy of the residual thyroid lobe is common in patients who develop hemiagenesis, thus supporting normal thyroid function [2]. The process was clearly seen in our patient as the right thyroid lobe was hypertrophied and was able to maintain a normal thyroid function, indicating a sign of a compensatory mechanism, as described in their paper.

Nevertheless, De Sanctis et al. noted that although compensatory hypertrophy helps sustain thyroid function at the beginning, thyroid dysfunction may emerge later if the remaining lobe cannot compensate for hormone production demands [4]. However, our patient had no thyroid dysfunction at seven years of follow-up. De Sanctis et al. stressed the importance of long-term follow-up in this condition, as thyroid insufficiency may become apparent in the future due to the limited functional reserve of the remaining lobe [7]. Although there was no thyroid dysfunction in our case, this study underscores the importance of regular and alert follow-up for the detection of any possible late-onset thyroid complication. Thus, although our patient’s condition has remained stable, it is important to remain vigilant for potential complications in patients with thyroid hemiagenesis.

Our case of thyroid hemiagenesis is notable for the presence of a paternal family history of hypothyroidism, a feature that is not commonly reported in the literature, where most cases lack a clear familial thyroid disease association. This may suggest a genetic predisposition to thyroid dysfunction, although no clear genetic mutation has been identified. In addition, although thyroid hemiagenesis is often associated with other structural abnormalities, such as renal or cardiovascular anomalies, our patient had no other congenital abnormalities, further supporting and highlighting the rarity and distinctiveness of her presentation.

## Conclusions

We presented a rare case of thyroid hemiagenesis in a 15-year-old female patient with a paternal family history of hypothyroidism, with agenesis of the left lobe, following the typical lateralization pattern. She had subclinical hypothyroidism, but achieved normalization of thyroid function on follow-up for more than seven years, reflecting adequate compensation by the right lobe. This case highlights the importance of regular follow-up in individuals with thyroid hemiagenesis and familial thyroid disease, given the potential for dynamic fluctuations in thyroid function with treatment, the risk of overcorrection, and the need to monitor for late-onset developments such as thyroid dysfunction or goiter. The familial nature suggests potential genetic or hereditary determinants of the clinical course of thyroid hemiagenesis. This case highlights the need for individualized care and lifelong follow-up to maximize thyroid function control throughout the patient’s life. Hence, further studies in the field, following such patients to assess the compensatory mechanism of a single lobe during increased physiological demand, are important.

## References

[1] Ruchala M, Szczepanek E, Szaflarski W (2010). Increased risk of thyroid pathology in patients with thyroid hemiagenesis: results of a large cohort case-control study. Eur J Endocrinol.

[2] Suzuki S, Midorikawa S, Matsuzuka T (2017). Prevalence and characterization of thyroid hemiagenesis in Japan: the Fukushima Health Management Survey. Thyroid.

[3] Szczepanek-Parulska E, Zybek-Kocik A, Wartofsky L, Ruchala M (2017). Thyroid hemiagenesis: incidence, clinical significance, and genetic background. J Clin Endocrinol Metab.

[4] Unar AA, Akhtar S, Ghaloo SK, Awan MO, Anjum S (2024). Thyroid hemiagenesis with compensatory hypertrophy of the remaining lobe: a case report. J Pak Med Assoc.

[5] De Sanctis V, Soliman AT, Di Maio S, Elsedfy H, Soliman NA, Elalaily R (2016). Thyroid hemiagenesis from childhood to adulthood: review of literature and personal experience. Pediatr Endocrinol Rev.

[6] Kaba E, Solak M (2023). Thyroid hemiagenesis. QJM.

[7] Avizov Khodak E (2024). [Thyroid hemiagenesis: a case report]. Harefuah.

